# Oleuropein Counteracts Both the Proliferation and Migration of Intra- and Extragonadal Seminoma Cells

**DOI:** 10.3390/nu14112323

**Published:** 2022-05-31

**Authors:** Sabrina Bossio, Anna Perri, Rocco Malivindi, Francesca Giordano, Vittoria Rago, Maria Mirabelli, Alessandro Salatino, Antonio Brunetti, Emanuela Alessandra Greco, Antonio Aversa

**Affiliations:** 1 Department of Experimental and Clinical Medicine, University of Catanzaro “Magna Græcia”, 88100 Catanzaro, Italy; sabrina.bossio@unicz.it (S.B.); anna.perri@unicz.it (A.P.); aversa@unicz.it (A.A.); 2 Department of Pharmacy, Health and Nutritional Sciences, University of Calabria, 87036 Rende, Italy; rocco.malivindi@unical.it (R.M.); francesca.giordano@unical.it (F.G.); vittoria.rago@unical.it (V.R.); 3 Department of Health Sciences, University of Catanzaro “Magna Græcia”, 88100 Catanzaro, Italy; maria.mirabelli@unicz.it (M.M.); salatino@unicz.it (A.S.); 4 Department Unicusano, Niccolò Cusano University, 00166 Rome, Italy; emanuela.greco@unicusano.it

**Keywords:** seminoma, oleuropein, NF-κB pathway, apoptosis, BAX

## Abstract

Recent and growing literature has reported that oleuropein (OLE), the main polyphenol in olive leaf extract, inhibits tumor cell proliferation and reduces the invasiveness properties of cancer cells; therefore, OLE may play a significant role in the development of new drugs for cancer treatment. These antineoplastic properties have been reported in many experimental cancer models, but the effect of OLE on seminoma cells is yet to be evaluated. In the present study, we demonstrate, for the first time, that OLE reduces cell viability in both intra- and extragonadal TCAM-2 and SEM-1 seminoma cells, respectively, in a dose-dependent manner. As shown by Western-blot analysis, OLE exposure reduced cyclin-D1 expression and upregulated p21^Cip/WAF1^, concomitantly affecting the upstream pathway of NF-κB, leading to the reduction of its nuclear content, thereby suggesting that OLE could modulate cell-cycle regulators by inhibiting NF-κB. Moreover, Annexin V staining revealed that OLE induced apoptosis in cancer cells and upregulated the pro-apoptotic factor BAX. Through wound-healing scratch and transmigration assays, we also demonstrated that OLE significantly reduced the migration and motility of TCAM-2 and SEM-1 cells, and downregulated the expression of TGFβ-1, which is known to be the main pro-fibrotic factor involved in the acquisition of the migratory and invasive properties of cancer cells. Collectively, our results indicate that OLE reduces seminoma cell proliferation, promotes apoptosis, and counteracts cell migration and motility. Further studies are needed to explore the molecular mechanisms underlying these observed effects.

## 1. Introduction

Testicular germ cell tumors (TGCTs) are rare cancers in childhood, adolescence, and young adulthood, whose incidence has been increasing in developed countries for at least four decades [1,2,3]. The reasons for this increase have not been completely elucidated yet, but it is reasonable to believe that environmental factors and irregular lifestyle may play an important role [3,4]. Seminoma accounts for about a third of all TGCT, and it is one of the most treatable cancers, with a survival rate of about 99% in early-stage disease [5]. Rarely, seminoma occurs outside of the gonads, with the mediastinum being the most common extragonadal location, because of defects from germ cell migration during embryogenesis. Mediastinal seminomas are indistinguishable from gonadal seminomas and are very sensitive to chemotherapy and radiation, as well as testicular seminomas [6]. Testicular seminoma is practically managed with surgical resection. In stage 1, a single therapy with carboplatin may be offered, whereas for more advanced disease, radiation and/or a combined chemotherapy may be used [5]. Although TGCT is an infrequent disease and is highly curable, it should be kept in mind that these affected young patients are exposed to the long-term consequences of chemotherapy-related toxicity or the long-term side-effects of radiotherapy, which negatively impact on their fertility and sexual activity [7,8]. In this respect, in the last few decades, growing literature has focused on the screening and identification of new therapeutic targets in cancer treatment, demonstrating the anticancer potential of natural agents from a variety of plants and their concomitant low toxicity. Many studies in experimental cancer models have demonstrated that polyphenols, which include a broad class of bioactive compounds contained in the Mediterranean diet, exert an anticancer activity by binding to and interacting with specific cellular targets [9].

Oleuropein (OLE), which is the major phenolic component of extra virgin olive oil [10], is gaining importance in the scientific community thanks to its therapeutic effects, including its anti-cancer function, so that the use of OLE can have a significant role in the development of new drugs [11,12,13]. The anticancer effects of OLE have been investigated in different in vitro models of reproductive system cancers [14,15,16,17,18,19,20]; however, to the best of our knowledge, there are no experimental studies on the effects of OLE, and more generally of polyphenols, in human testicular cancer. Therefore, in this study, we explored the anticancer effects of OLE in in vitro models of both intra- and extragonadal seminoma.

## 2. Materials and Methods

### 2.1. Cell Culture and Treatment

The testicular cancer cell lines, SEM-1 and TCAM-2 (a kind gift from Prof. A.L. Epstein, CA, USA), and HepG2 human hepatoma cells (American Type Culture Collection, Manassas, VA, USA) were cultured in an RPMI-1640 culture medium, supplemented with 10% fetal bovine serum, 1% glutammine, and 1 mg/mL penicillin/streptomycin (Sigma Aldrich, Milano, Italy). All of the cell lines were cultured in 100 mm dishes, and were kept incubated at 37 °C in an atmosphere of 5% CO_2_. All of the experiments were performed after 12 h of cell synchronization in serum-free media (SFM). The OLE was obtained from Sigma Aldrich (cat. N. O8889, St. Louis, MO, USA), and was dissolved in double-distilled water and diluted in RPMI-1640 media before use.

### 2.2. Cell Viability Assay

Cell viability was determined using the 3-(4,5-dime-thylthiazol-2-yl)-2,5-diphenyltetrazolium (MTT) assay. Briefly, the cells were implanted in 96-well plates at a density of 3 × 10^4^ for SEM-1, 4 × 10^4^ for TCAM-2, and 2 × 10^4^ for HepG2, and were synchronized in SFM for 12 h. The cells were exposed to treatments with increasing doses of OLE (15–200 μM) for 48 h, as this experimental time equalized the cell division length of both types of seminoma cells and HepG2 [21]. Eight replicates were performed for each sample. Then, 20 µL of MTT (5 mg/mL) was added to the cell media, and after 4 h of incubation, isopropanol (200 µL) was added to each well and the optical density was measured at 570 nm using a Beckman Coulter microplate reader.

### 2.3. Annexin V Assay

The cells were seeded in six multi-well plates and were allowed to attach overnight. The cells were synchronized in SFM for 12 h, and then treated with OLE for 48 h. Before collecting, the cells were rinsed with warm phosphate buffered saline (PBS) and harvested by enzymatic digestion (0.125% trypsin-EDTA solution, Sigma Aldrich) for 5 min. Then, the cells were centrifuged at 1000 rpm for 5 min at room temperature. The FITC-Annexin V Apoptosis Detection kit (BD Bioscience, Heidelberg, Germany) was used according to the manufacturer’s protocol. Briefly, the cell suspension was washed twice with PBS and was resuspended in 1× binding buffer. FITC-annexin (5 µL) and propidium iodide (5 µL) were added to 100 µL of the cell suspension, which was then incubated for 15 min at room temperature in the dark. Finally, 400 µL of binding buffer were added, and the cells were analyzed by flow cytometry using FACSCanto II (BD Bioscience, Heidelberg, Germany) and FACSDiva software v. 6.1.3 (BD Bioscience, Heidelberg, Germany). For each condition, 10,000 cells/event were analyzed.

### 2.4. Protein Extraction and Western Blot Analysis

The cells were grown in 100 mm dishes to 70–80% confluence and were treated with media containing OLE. After 48 h of treatment, the cell monolayers were washed with cold PBS and were solubilized in a RIPA buffer (Cell Signaling Technology, Danvers, MA, USA), plus phenylmethanesulfonyl fluoride (PMSF) at 1 mM concentration. The cell lysates were quantified spectrophotometrically using the Bio-Rad Bradford Assay (Bio-Rad Laboratories, Hercules, CA, USA). All of the samples were loaded on a 10% or 15% SDS–polyacrylamide gel; transferred to a nitrocellulose membrane; and probed with antibodies directed against cyclin D1 (CD1) (M3642 Dako Agilent), p21^Cip/WAF1^ (sc-6246), NFκB (sc-8008), Bax (sc-7480), TGFβ-1 (sc-146), p-IKBα (sc-101713), and tot-IKBα (sc-373893) (Santa Cruz Biotechnology, Santa Cruz, CA, USA). As the internal control, all of the membranes were probed with anty-glyceraldehyde-3-phosphate dehydrogenase, GAPDH (sc-47724), and Lamin-B (sc-6217) antibodies (Santa Cruz Biotechnology). The antigen–antibody complex was detected through incubation of the membranes for 1 h at room temperature with peroxidase-coupled goat anti-mouse, anti-rabbit, or anti-goat IgG and revealed using the enhanced chemiluminescence system (Santa Cruz Biotechnology). The blots were then exposed to film (Santa Cruz Biotechnology). The intensity of bands representing the relevant proteins was measured using Image J densitometry scanning software [22].

For nuclear and cytosolic extracts, the cells were centrifuged at 1000 rcf for 5 min at room temperature, washed with cold PBS, and centrifuged at 2500 rcf for 5 min at 4 °C. The cell pellets were resuspended in a cytosolic buffer (10 mM HEPES pH 7.9, 10 mM KCl, 0.1 mM EDTA, 1 mM DTT, and 0.5 mM PMSF), incubated for 15 min at 4 °C on wheel and then centrifuged at 7000 rcf for 5 min at 4 °C. The resulting nuclear pellet was resuspended in a nuclear buffer (20 mM HEPES pH 7.9, 0.4 M NaCl, 1 mM EDTA, 1 mM EGTA, 1 mM DTT, and 1 mM PMSF), incubated for 15 min at 4 °C on wheel, and then centrifuged at 7000 rcf for 5 min at 4 °C. The supernatants represent the nuclear extracts. Proteins of the nuclear fractions were determined using a Bio-Rad Bradford Assay (Bio-Rad Laboratories). Equal amounts of proteins (30 µg) were resolved using 10% SDS–polyacrylamide gel electrophoresis, transferred to nitrocellulose membranes, and probed against antibodies to NF-κB (p65) (sc-8008, Santa Cruz Biotechnology).

### 2.5. Wound-Healing Scratch Assay

SEM-1 and TCAM-2 cells were grown to confluence in regular media, and then maintained in SFM for 12 h. The monolayers were scratched as previously described [23] and were treated with OLE. Then, wound healing was photographed at 24 h at 4× magnifications using phase-contrast microscopy.

### 2.6. Transmigration Assay

Testicular cancer cell lines were treated as indicated and were placed in the upper compartments of a Boyden chamber (8 mm membranes; Corning Costar, NY, USA), as previously reported [24]. After 12 h, the migrated cells were fixed and stained with 4′,6-diamidino-2-phenylindole (DAPI). Migration was quantified by viewing five separate fields per membrane at 10× magnification and were expressed as the mean number of migrated cells.

### 2.7. Statistical Analysis

Data were analyzed by Student’s *t* test using the GraphPad Prism 8.3.0 (GraphPad Software, Inc., San Diego, CA, USA). *p* < 0.05 was considered as statistically significant.

## 3. Results

### 3.1. OLE Reduces Seminoma Cells Viability, by Promoting Apoptosis

**Figure 1:**
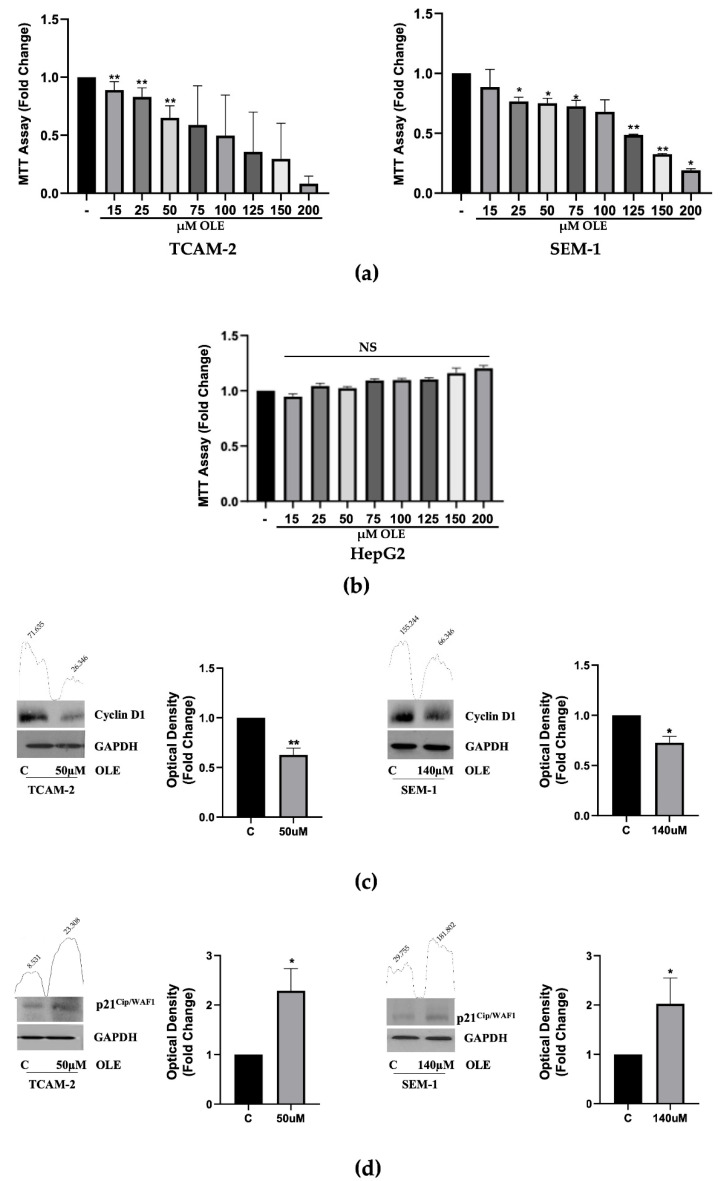
(**a**,**b**) MTT assay in testicular cancer cells and HepG2 cells. TCAM-2 and SEM-1 cells were either left untreated or treated with increasing doses (15–200 µM) of OLE for 48 h. Cell proliferation is expressed in fold changes ± standard deviations (SDs) with respect to the basal conditions and is representative of three independent experiments, each performed in eight replicates. Statistical significance was considered at * *p* < 0.05; ** *p* < 0.001 NS = not significant. Statistical comparisons were drawn between groups using a one-tailed *t*-test. (-) = control. (**c**) Immunoblot showing the CD1 protein expression in TCAM-2 and SEM-1 cells exposed for 48 h to OLE (50 µM TCAM-2; 140 μM SEM-1). GAPDH was used as a loading control. The histograms represent the mean ± SD of three separate experiments (each performed in triplicate) in which band intensities were evaluated as the optical density and are represented as fold changes for treated vs. untreated cells normalized for the loading control. ** *p* < 0.001 and * *p* < 0.05 for treated vs. untreated cells. (**d**) Protein expression of p21^Cip/WAF1^ in TCAM-2 and SEM-1 cells exposed for 48 h to OLE (50 µM TCAM-2; 140 μM SEM-1). GAPDH was used as a loading control. As in (**c**), the histograms represent the mean ± SD of three separate experiments (each performed in triplicate), in which the band intensities were evaluated as the optical density and are represented as fold changes for treated vs. untreated cells normalized to the loading control. The numbers on the peaks reported on the top of each blot represent the size of the corresponding slot as a percentage of the total size of the two slots in each condition. * *p* < 0.05 for treated vs. untreated cells.

A cell viability assay was used to investigate the cytotoxic effects of OLE exposure in the testicular cancer cell lines SEM-1 and TCAM-2. To this end, the cells were synchronized in SFM for 12 h, and then treated with increasing doses of OLE (15–200 μM) for 48 h. MTT assay results demonstrated that OLE exposure promoted a significant dose-dependent reduction of cell viability (Figure 1a), with a half maximal inhibitory concentration (IC_50_) estimated as 140 µM for SEM-1 and 50 µM for TCAM-2 (Table 1). Therefore, these concentrations were used for all of the successive experiments reported in this paper. Furthermore, as it has been demonstrated that liver is the main organ deputed to phenolic metabolism [25], we investigated the cytotoxicity of OLE in HepG2 cells. The MTT results showed that exposure to increasing doses of OLE (15–200 μM) for 48 h did not affect HepG2 cell viability (Figure 1b).

Next, we investigated whether OLE was able to affect the protein expression of two key regulators of the cell cycle, CD1 and p21^Cip/WAF1^. Western blot analyses of these proteins showed that OLE exposure promoted a significant downregulation of CD1, with a concomitant upregulation of p21^Cip/WAF1^ expression (Figure 1c,d), suggesting that OLE could reduce seminoma cell proliferation by affecting the cell-cycle progression.

**Figure 2:**
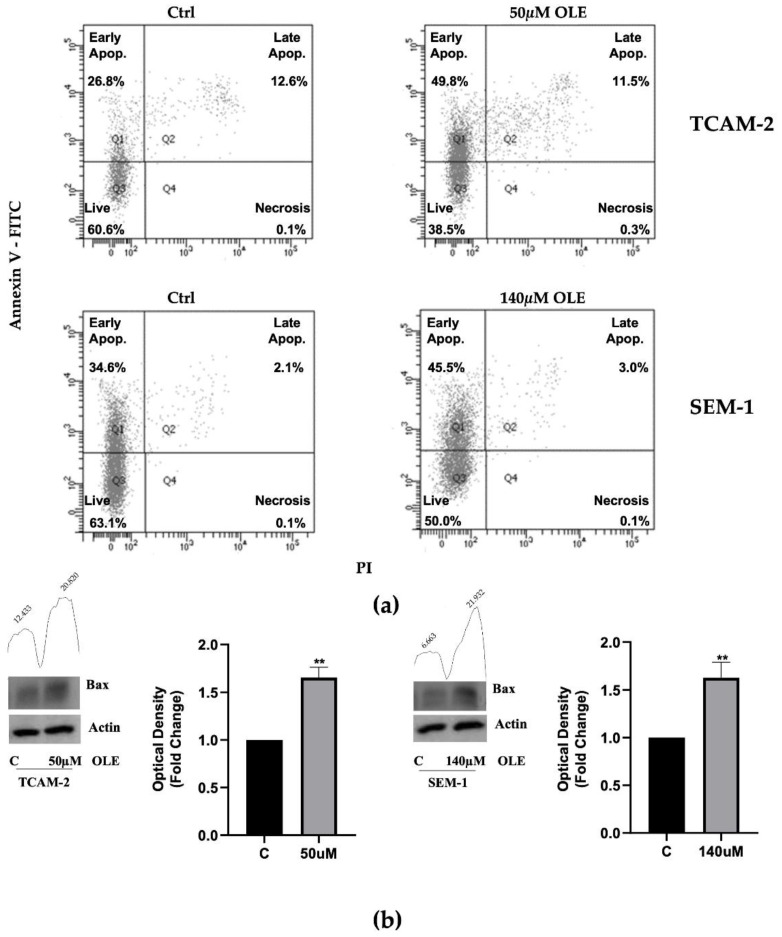
(**a**) An annexin V/PI assay was performed on SEM-1 and TCAM-2 cells using flow cytometry analysis. The following percentages of cells are represented in the figure: viable cells in the bottom left (PI/Annexin −/−; Q_3_), early apoptotic cells in the top left (PI/Annexin −/+; Q_1_), late apoptotic cells in the top right (PI/Annexin +/+; Q_2_), and necrotic cells in bottom right (PI/Annexin +/−; Q_4_). (**b**) Immunoblotting showing Bax protein expression in TCAM-2 and SEM-1 cells exposed for 48 h to 50 µM and 140 µM of OLE, respectively. β-Actin was used as the loading control. The histograms represent the mean ± SD of three separate experiments (each performed in triplicate) in which the band intensities were evaluated as the optical density and are represented as fold change for treated vs. untreated cells normalized for the loading control. The numbers on the peaks reported on the top of each blot represent the size of the corresponding slot as a percentage of the total size of the two slots in each condition. ** *p* < 0.001 treated vs. untreated cells.

To explore whether apoptosis could be involved in the anti-proliferative effect of OLE, an Annexin V-FITC/PI assay was performed. To this end, SEM-1 and TCAM-2 cells were treated with OLE for 48 h. As reported in Figure 2a, the percentage of early apoptotic cells was 45.5% for SEM-1 cells and 49.8% for TCAM-2 cells. A group of cells in late apoptosis was also observed (3% for SEM-1 and 11.5% for TCAM-2 cells) with a few cells almost in the necrosis phase. In the untreated controls, the cells remained viable with a percentage of 63.1% for the SEM-1 cells and 60.6% for the TCAM-2 cells (Figure 2a).

Next, we investigated whether OLE exposure could affect the protein levels of the pro-apotpotic marker Bax, whose expression in seminoma correlates closely with the apoptotic index and seems to be linked to a favorable outcome [26]. As reported in Figure 2b, the Western blot analyses demonstrated that in both TCAM-2 and SEM-1 cells, OLE significantly augmented the Bax expression, suggesting that the pro-apoptotic effect of OLE in these cell lines could occur by enhancing the expression of Bax.

### 3.2. In Seminoma Cells, OLE Reduces NF-κB Nuclear Translocation by Affecting Its Upstream Pathway

**Figure 3:**
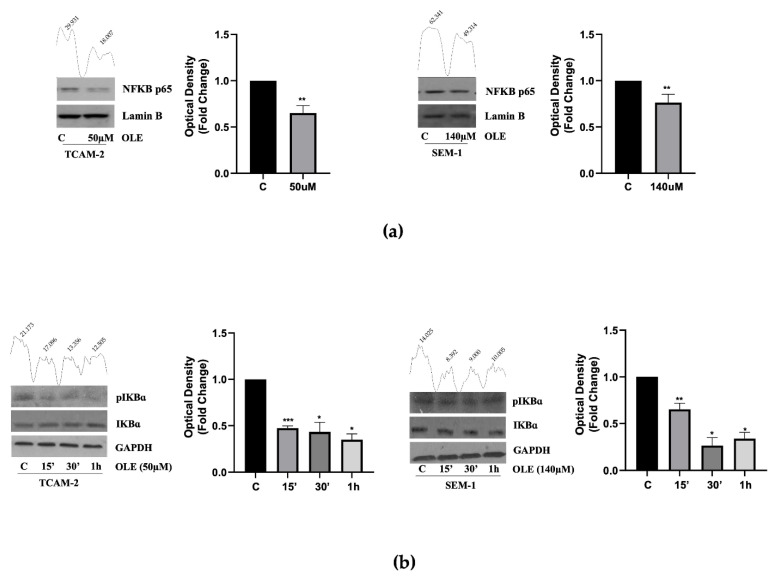
(**a**) The Western blot analysis of the nuclear protein extracts shows that both in TCAM-2 and SEM-1 cells, there was a significant reduction of NF-κB nuclear translocation after the exposure of OLE (50 µM for TCAM-2; 140 µM for SEM-1). Lamin β was used as the loading control. The histograms represent the mean ± SD of three separate experiments (each performed in triplicate) in which the band intensities were evaluated as the optical density and are represented as fold change for the treated vs. untreated cells normalized for the loading control. ** *p* < 0.001 treated vs. untreated cells. (**b**) TCAM-2 and SEM-1 cell lines were treated at 15 min, 30 min, and 1 h with OLE (50 µM for TCAM-2; 140 µM for SEM-1). The Western blot analysis of the total protein extracts shows a down-regulation of pIkB-α in TCAM-2 and SEM-1 cells. GAPDH was used as the loading control. As in (**a**), the histograms represent the mean ± SD of three separate experiments in which bands intensities were evaluated as the optical density and are represented as fold change for the treated vs. untreated cells normalized for the loading control. The numbers on the peaks reported on the top of each blot represent the size of the corresponding slot as a percentage of the total size of the two slots in each condition. * *p* < 0.05, ** *p* < 0.001, *** *p* < 0.0001 treated vs. untreated cells.

Several studies reported that the transcription factor NF-κB modulates the transcriptional activity of different target genes, including CD1 and p21^Cip/WAF1^ [27,28], and that in some in vitro cancer models, OLE reduces the NF-κB expression, leading to the inhibition of tumor cell growth [29,30]. Based on this evidence, we first investigated the nuclear content of NF-κB in SEM-1 and TCAM-2 cells, either untreated or treated with OLE. Interestingly, the Western blot analyses showed that in both cell lines, OLE exposure significantly reduced nuclear translocation of the NF-κB protein (Figure 3a).

Therefore, we investigated whether these results were strictly dependent on the OLE-modulation of the IkB kinase complex (IKK), a key upstream regulator of NF-κB activation. The time course experiments, in this context, revealed that cell treatment with OLE protects the inhibitory IkB-α subunit from phosphorylation and cleavage, thereby preventing the nuclear translocation of active NF-κB (Figure 3b). Collectively, these results suggest that OLE could modulate both CD1 and p21^Cip/WAF1^ in a transcriptional-dependent manner via the inhibition of the NF-κB pathway.

### 3.3. OLE Counteracts Seminoma Cells’ Migratory Capability

**Figure 4:**
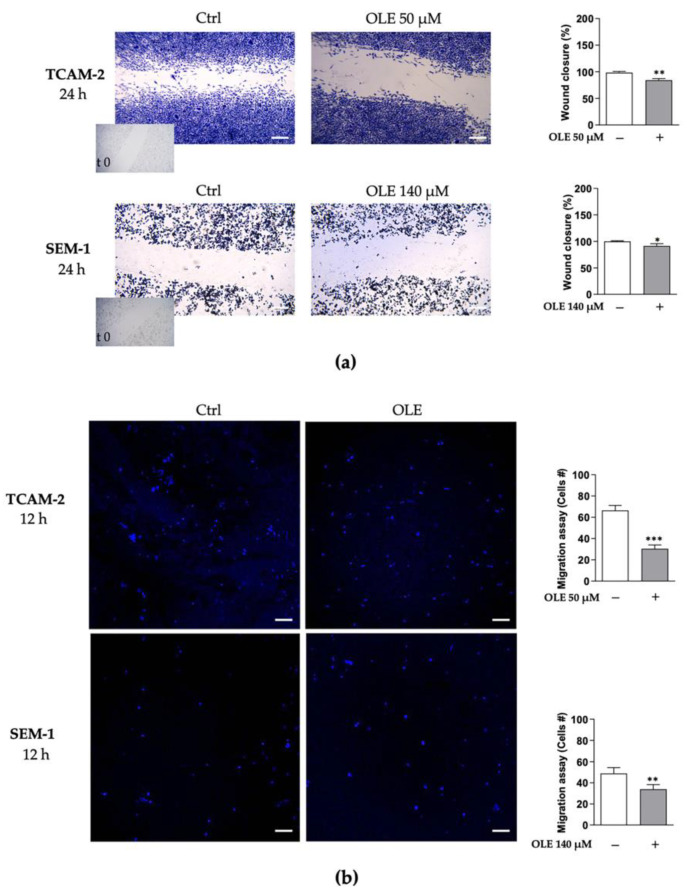
(**a**) A wound-healing assay was performed as described previously [23]. Briefly, cell mono-layers were scraped, and the cells were treated with OLE (50 µM for TCAM-2; 140 µM for SEM-1) and were monitored for 24 h. Then, in order to assess the differences in the migratory properties with respect to the control conditions, intragonadal and extragonadal seminoma cells were fixed and stained with Comassie Brillant Blue. Pictures were taken at 4× magnification using phase-contrast microscopy and are representative of three independent experiments. The percentage of wound clousure was quantifyed over 24 h using ImageJ. Scale bar: 100 µm, * *p* < 0.05, ** *p* < 0.001 treated vs. untreated cells; (**b**) transmigration assay. Cells treated as indicated in (**a**) were placed in the upper compartments of a Boyden chamber (8 mm membranes; Corning Costar, NY, USA). The bottom well contained regular full media. After 12 h, the migrated cells were fixed and stained with DAPI. Migration was quantified by viewing five separate fields per membrane at 10× magnification, and are expressed as the mean number of migrated cells. Data represent three independent experiments assayed in triplicate. Scale bar: 50 µm, ** *p* < 0.001 and *** *p* < 0.0001 treated vs. untreated cells.

Notably, different in vitro studies have demonstrated that OLE exerts anti-migratory effects in various types of cancer by affecting different pathways [31]. In order to investigate the ability of OLE to counteract the migratory properties of TCAM-2 and SEM-1 cells, we employed two approaches to study the migratory capacity of these cells: wound-healing and transwell migration assays (Figure 4).

**Figure 5:**
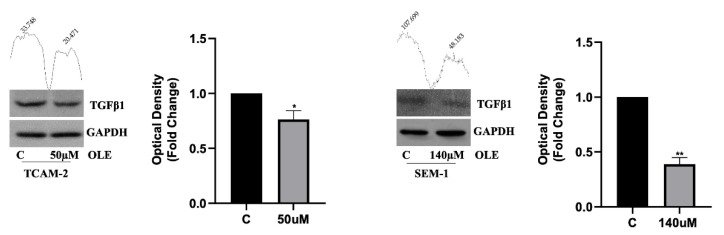
Western blot analysis shows a down-regulation in both TCAM-2 and SEM-1 cells after OLE treatment (50 µM for TCAM-2; 140 µM for SEM-1) for 48 h. GAPDH was used as loading control. The histograms represent the mean ± SD of three separate experiments (each performed in triplicate) in which bands intensities were evaluated as optical density and represented as fold change for treated vs. untreated cells normalized for the loading control. The numbers on the peaks reported on the top of each blot represent the size of the corresponding slot as a percentage of the total size of the two slots in each condition. * *p* < 0.05, ** *p* < 0.001 treated vs. untreated cells.

Our results revealed that OLE treatment significantly counteracted wound closure (Figure 4a), as well as cell motility (Figure 4b), compared with the untreated cells. Interestingly, we observed that the anti-migratory effects promoted by OLE exposure were more evident in TCAM-2 cells than in SEM-1 cells. Indeed, in treated TCAM-2 cells, there was a reduction in cell migration rate and cellular motility of −14.27 ± 2.065 and −36.00 ± 2.631, respectively (difference means ± standard error of the mean (s.e.m.); *p* < 0.05), compared with the untreated cells. Instead, in the treated SEM-1 cells, we observed decreased cell migration and motility in the amounts of −8.503 ± 2.440 (difference means ± s.e.m.; *p* < 0.05) and of −14.80 ± 3.184 (difference means ± s.e.m.; *p* < 0.001), respectively, compared with the control cells. Furthermore, in line with the above reported results, we observed that in both TCAM-2 and SEM-1 cells, OLE decreased the expression of the key pro-fibrotic marker, TGF-β1 (Figure 5), suggesting that OLE could counteract the epithelial-to-mesenchymal transition (EMT) process by affecting TGF-β1 protein production.

## 4. Discussion

In this study, we have demonstrated, for the first time, that in both intra and extragonadal seminoma cell lines, OLE reduces tumor cell growth by promoting apoptosis and counteracting cellular motility and migration. Seminomas represent the most common histological subtype of TCGTs. Although they are rare, respond well to chemo- and radiotherapy, and show high survival rates [32], it should be kept in mind that the long-term side effects of platin-based chemotherapy, as well as of radiotherapy, may significantly decrease the quality of life of very young patients. Consequently, less toxic treatment strategies are needed for these individuals. The findings emerging from our study are in agreement with in vitro and in vivo evidence showing that OLE may induce antineoplastic effects by affecting different oncogenic targets, including transcription factors, cytokines, adhesion molecules, growth factors, etc., making it a promising potential natural therapeutic in the treatment of a variety of cancers [33]. Although the antineoplastic activities of OLE have been demonstrated in different in vitro cancer models, mainly in breast cancer cells, to the best of our knowledge, there are no studies that have investigated the antineoplastic effects of OLE in seminoma cells.

We found that OLE negatively affects both TCAM-2 and SEM-1 cell proliferation, although in the extra-gonadal germ cell line, SEM-1, the anti-growth effect was achieved by a higher dose of OLE, suggesting that these cells are less responsive than TCAM-2. It has been reported [21] that SEM-1 cells show intermediate characteristics between seminoma and non-seminoma, highlighting that, although SEM-1 cells display morphologic and growth parameters similar to TCAM-2, they could represent a tumor model with more aggressive clinical features [21]. Our results, in the present study, reveal that in both TCAM-2 and SEM-1 cells, the antiproliferative effect of OLE was accompanied by modulation of the key cell cycle regulatory proteins, CD1 and p21^Cip/WAF1^. At the same time, we found that cell exposure to OLE affected the NF-κB activation cascade, leading to a significant reduction of NF-κB nuclear translocation. It is well known that NF-κB has a pro-survival activity, as it can stimulate and inhibit, in a transcriptional-dependent manner, CD1 and p21^Cip/WAF1^ DNA promoters, respectively [27,28]. Our findings fit well with previous data, demonstrating that NF-κB represents a molecular target through which OLE promotes antineoplastic effects. In this regard, it has been reported that in MCF-7 breast cancer cells, exposure to OLE decreased the expression of CD1 and NF-κB and increased the expression of p21^Cip/WAF1^, preventing cancer cell proliferation [34]. Similarly, other in vitro and vivo studies showed that in both estrogen receptor negative breast cancer cells and in MCF-7 xenograft models, OLE reduced the expression of NF-κB by regulating its cascade activation, leading to tumor cell apoptosis [29,30]. In addition, it has been demonstrated that in cervical cancer cells, an olive leaf extract rich in OLE counteracted cancer cell proliferation by activating p21^Cip/WAF1^ in a transcriptional-dependent manner, and reducing the nuclear recruitment of NF-κB on its DNA responsive elements [28]. Therefore, although no functional studies were performed in our work, we can speculate that the intranuclear decrease in NF-κB induced by OLE could contribute to modulating the CD1 and/or p21^Cip/^WAF1 expression, which may represent a mechanistic explanation for the observed anti-tumor growth effects of OLE.

In addition to the antiproliferative effects, the annexin-V staining and Western blot results obtained in the present study show that in both TCAM-2 and SEM-1 cells, exposure to OLE promoted cell apoptosis by concomitantly increasing the pro-apoptotic potential in these cells through the overexpression of BAX. These pro-apoptotic effects of OLE are in line with previous research findings, indicating that OLE may induce cancer cell death by interfering with the intrinsic and extrinsic apoptosis pathways [33]. In this regard, a significant correlation between BAX-positive seminoma cells and the apoptotic index has been reported in a study showing that Bax seems to play a role in modulating apoptosis in human seminoma, and this would explain, at least in part, the favorable outcome of this tumor, as observed in some instances [26]. In addition, in this context, it has been reported that in lung adenocarcinoma cells, OLE increased the Bax/Bcl-2 ratio and activation of caspase-9 and -3, leading to cell apoptosis [35]. Similarly, OLE reduced neuroblastoma cell proliferation, concomitantly promoting apoptosis, through the increase of both Bax and p53 [36]. Therefore, although further studies are needed to better clarify the molecular mechanism(s) involved in these phenomena, it is tempting to speculate that, in seminoma cells, the anti-proliferative effects of OLE are linked to the activation of apoptosis and that the upregulation of Bax could have an important role in the observed anti-tumor growth effect of OLE. Interestingly, our motility assay results revealed that OLE significantly reduced TCAM-2 and SEM-1 cell migration and motility, although these effects were more evident in TCAM-2 cells. These results are in agreement with those previously reported in the literature, showing that OLE efficaciously counteracts the migratory properties of cancer cells by affecting different molecular pathways [33]. Moreover, we observed that OLE exposure significantly downregulated the TGF-β1 protein expression, a master regulator of EMT, which represents the primary step in metastatic dissemination, by promoting the acquisition of the migratory and invasive properties of cancer cells [37,38,39]. It is well known that the TGF-β1 signaling pathway activation promotes the phenotypic changes of epithelial cells, with transition to a mesenchymal phenotype, and upregulates the expression of the metalloproteinases involved in the degradation of the extracellular matrix, thereby favoring cell migration and invasion [40]. Recently, an overexpression of the pituitary tumor-transforming gene 1 (PTTG1) and its transcriptional target matrix-metalloproteinase-2 have been reported in TCAM-2 and SEM-1 cells, suggesting that PTTG1 might play a role in the invasiveness and metastasis of cancer cells [39]. Additionally, the involvement of PTTG1 in the proliferation and invasive potential of different human cancer cells has also been suggested through the regulation of the TGF-β1 signaling pathway [40]. Therefore, studies should be performed to investigate whether the impairment of seminoma cell motility and migration by OLE could involve PTTG1 expression.

We are aware that the results of the present study are descriptive in nature and the functional mechanism(s) underlying the observed effects remain unclear. By taking our findings into a clinical scenario, it could be proposed that young people with at-risk behaviors for reproductive injury [41] could benefit from dietary intervention and supplementation with OLE and other polyphenols from olive leaves.

## Figures and Tables

**Table 1:** IC_50_ value of OLE in TCAM-2 and SEM-1 cells derived from the MTT assay.

Cell Lines	IC_50_ Value (μM)	95% Confidence Interval
TCAM-2	50	62.25–108.5
SEM-1	140	169.5–32.7

The concentration of OLE required to inhibit 50% cell viability over 48 h (IC_50_ value) was calculated by analyzing the relationship, shown in Figure 1a, between increasing doses of OLE and percent (%) inhibitions using GraphPad Prism 8.3.0 (GraphPad Software, Inc., San Diego, CA, USA).

## Data Availability

Not applicable.
