# Correction: Bossio et al. Oleuropein Counteracts Both the Proliferation and Migration of Intra- and Extragonadal Seminoma Cells. *Nutrients* 2022, *14*, 2323

**DOI:** 10.3390/nu18101632

**Published:** 2026-05-21

**Authors:** Sabrina Bossio, Anna Perri, Rocco Malivindi, Francesca Giordano, Vittoria Rago, Maria Mirabelli, Alessandro Salatino, Antonio Brunetti, Emanuela Alessandra Greco, Antonio Aversa

**Affiliations:** 1 Department of Experimental and Clinical Medicine, University of Catanzaro “Magna Græcia”, 88100 Catanzaro, Italy; sabrina.bossio@unicz.it (S.B.); anna.perri@unicz.it (A.P.); aversa@unicz.it (A.A.); 2 Department of Pharmacy, Health and Nutritional Sciences, University of Calabria, 87036 Rende, Italy; rocco.malivindi@unical.it (R.M.); francesca.giordano@unical.it (F.G.); vittoria.rago@unical.it (V.R.); 3 Department of Health Sciences, University of Catanzaro “Magna Græcia”, 88100 Catanzaro, Italy; maria.mirabelli@unicz.it (M.M.); salatino@unicz.it (A.S.); 4 Department Unicusano, Niccolò Cusano University, 00166 Rome, Italy; emanuela.greco@unicusano.it

In the original publication [1], there was a mistake in “Figure 1C” as published. Specifically, due to an error by the art department during figure assembly, the same GAPDH blot used for TGF-β1 in the SEM-1 cells in Figure 5 was mistakenly used for cyclin D1 in the TCAM-2 cell line. The original blot for cyclin D1 in the TCAM-2 cells has now been restored. The corrected version of Figure 1 appears below. The authors confirm that the scientific conclusions are not affected by this correction. This correction has been approved by the Academic Editor. The original publication has also been updated.

## Figures and Tables

**Figure 1:**
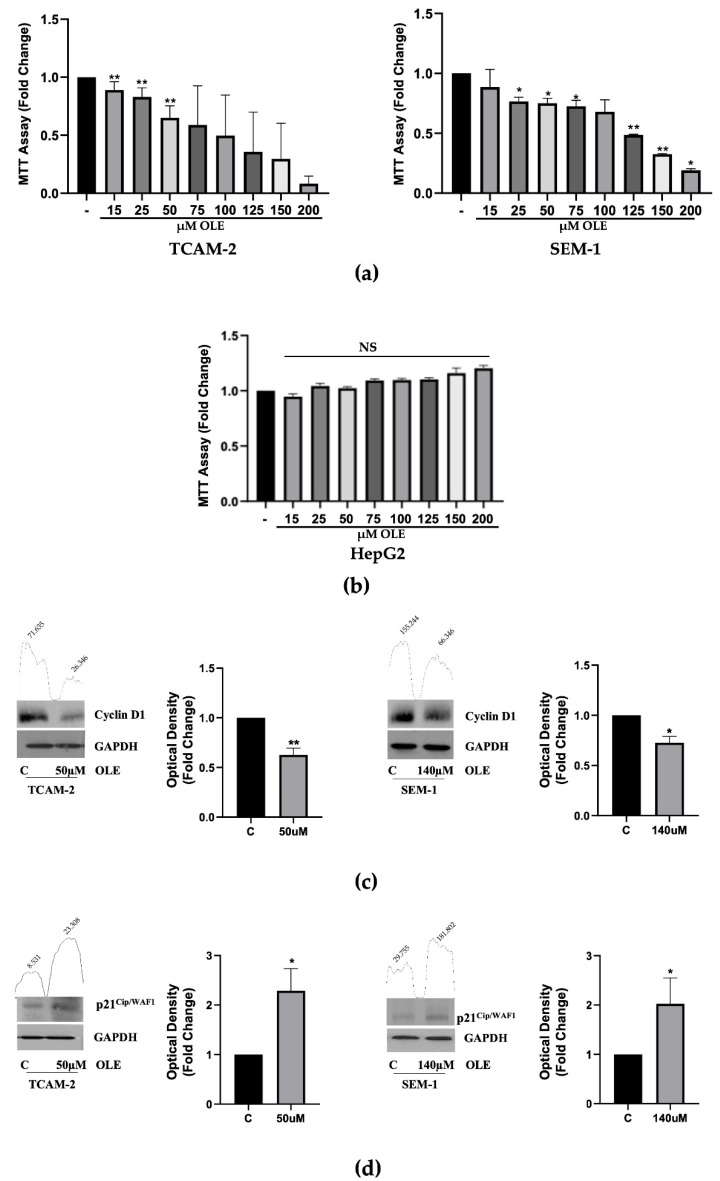
(**a**,**b**) MTT assay in testicular cancer cells and HepG2 cells. TCAM-2 and SEM-1 cells were either left untreated or treated with increasing doses (15–200 µM) of OLE for 48 h. Cell proliferation is expressed in fold changes ± standard deviations (SDs) with respect to the basal conditions and is representative of three independent experiments, each performed in eight replicates. Statistical significance was considered at * *p* < 0.05; ** *p* < 0.001 NS = not significant. Statistical comparisons were drawn between groups using a one-tailed *t*-test. (-) = control. (**c**) Immunoblot showing the CD1 protein expression in TCAM-2 and SEM-1 cells exposed for 48 h to OLE (50 µM TCAM-2; 140 μM SEM-1). GAPDH was used as a loading control. The histograms represent the mean ± SD of three separate experiments (each performed in triplicate) in which band intensities were evaluated as the optical density and are represented as fold changes for treated vs. untreated cells normalized for the loading control. ** *p* < 0.001 and * *p* < 0.05 for treated vs. untreated cells. (**d**) Protein expression of p21^Cip/WAF1^ in TCAM-2 and SEM-1 cells exposed for 48 h to OLE (50 µM TCAM-2; 140 μM SEM-1). GAPDH was used as a loading control. As in (**c**), the histograms represent the mean ± SD of three separate experiments (each performed in triplicate), in which the band intensities were evaluated as the optical density and are represented as fold changes for treated vs. untreated cells normalized to the loading control. The numbers on the peaks reported on the top of each blot represent the size of the corresponding slot as a percentage of the total size of the two slots in each condition. * *p* < 0.05 for treated vs. untreated cells.
